# Improvement in bladder volume reproducibility using A‐mode portable ultrasound bladder scanner in moderate‐hypofractionated volumetric modulated arc therapy for prostate cancer patients

**DOI:** 10.1002/acm2.13546

**Published:** 2022-02-02

**Authors:** Shingo Ohira, Riho Komiyama, Naoyuki Kanayama, Kayo Sakai, Takero Hirata, Kento Yoshikata, Yoshihiro Ueda, Masayoshi Miyazaki, Masashi Nakayama, Masahiko Koizumi, Koji Konishi

**Affiliations:** ^1^ Department of Radiation Oncology Osaka International Cancer Institute Osaka Japan; ^2^ Department of Medical Physics and Engineering Osaka University Graduate School of Medicine Suita Japan; ^3^ Nursing Department Osaka International Cancer Institute Osaka Japan; ^4^ Department of Radiation Oncology Osaka University Graduate School of Medicine Suita Japan; ^5^ Department of Urology Osaka International Cancer Institute Osaka Japan

**Keywords:** bladder, portable ultrasound bladder scanner, prostate cancer

## Abstract

**Purpose:**

This study introduced an A‐mode portable ultrasound bladder scanner, the Lilium® α‐200 (here after Lilium; Lilium Otsuka, Kanagawa, Japan), for the treatment of prostate cancer patients with hypofractionated volumetric modulated arc therapy to improve the reproducibility of bladder volume (BV).

**Materials and methods:**

Thirty patients were advised to maintain full BV prior to computed tomography (CT) simulation and daily treatment. Among these, the BV of 15 patients was measured using Lilium until a BV of 80% in the simulation was achieved (with the Lilium group). Daily cone‐beam CT (CBCT) was performed for treatment. The correlation between BV measured by CBCT and Lilium was assessed. The differences in the BV and dosimetric parameters of the bladder in the CBCT versus planning CT were compared between the groups with and without Lilium.

**Results:**

There was a significantly strong relationship (*r* = 0.796, *p* < 0.05) between the BVs measured using CBCT and Lilium. The relative BV ratios to simulation CT < 0.5 and > 2 were observed in 10.3% and 12.7%, respectively, of treatment sessions without Lilium group, while these ratios were 1% and 2.8%, respectively, in the Lilium group. The mean absolute difference in the range of V_30Gy_ to V_40Gy_ without Lilium sessions was significantly larger (*p* < 0.05) than that in the Lilium group.

**Conclusion:**

The use of the A‐mode portable ultrasound bladder scanner significantly improved the reproducibility of the BV, resulting in few variations in the dosimetric parameters for the bladder.

## 1 INTRODUCTION

External beam radiotherapy is an effective treatment option for patients with prostate cancer. Recent irradiation techniques, such as volumetric modulated arc therapy (VMAT), in conjunction with image‐guided radiotherapy, can generate a conformal dose to the target while minimizing exposure to the surrounding organs at risk (OARs) with high accuracy.^1^, ^2^, ^3^ Using these techniques, moderate‐ (2.2–4 Gy per fraction) or ultra‐hypofractionated (> 4 Gy) radiotherapy is increasingly applied in patients with prostate cancer owing to its superiority in terms of biological effect for improving therapeutic ratio compared with the standard fractionated radiotherapy (1.8–2 Gy),^4^ shortened treatment time as an alternative to surgery,^5^ and circumstance of COVID‐19 pandemic.^6^

Patients are usually advised to drink a fixed volume of water at a fixed time before the treatment simulation to acquire computed tomography (CT) images of patients with comfortably full bladders. The advantage of maintaining a full bladder is to decrease the radiation exposure to the bladder and small bowels.^7^ However, Chen observed significant variation in the daily bladder volume (BV) on the daily cone‐beam CT (CBCT) compared with the CT images used for treatment planning for prostate cancer radiotherapy, and the dosimetric parameters of the bladder changed proportionately depending on BV variations.^8^ Because hypofractionated radiotherapy may induce side effects,^4^ maintaining a constant BV during the treatment course is challenging.

A new portable ultrasound bladder scanner (A‐mode), named Lilium® α‐200 (here after Lilium; Lilium Otsuka, Kanagawa, Japan),^9^ was developed, and the device could monitor and record the BV continuously through a small probe. Majima et al. has shown the feasible accuracy of the BV measurement using the Lilium in the multicenter study.^10^ Although the assessment of BV before daily radiotherapy using a CT scanner is time‐consuming, and patients need to be exposed to radiation, the A‐mode device may act as a screening tool of the BV prior to performing CBCT scans. In addition, the bladder scanner using the A‐mode is more cost‐effective than that using the B‐mode, which is introduced in clinical radiotherapy practice to assess the BV.^11^, ^12^ The Lilium has the potential to reduce the daily variation of BV with the minimal workload in radiotherapy for patients with prostate cancer.

This study assessed the relationship between the daily BV measured by CBCT and Lilium, as well as to investigate whether the use of Lilium improves the reproducibility of BV during the treatment course in prostate cancer patients.

## 2 MATERIALS AND METHODS

### 2.1 Simulation and treatment planning

The Lilium was introduced in the clinical practice to reduce the daily BV variation in pelvic radiotherapy, and BV was measured for patients with prostate, bladder, and postoperative prostate cancer. This retrospective study was approved by the ethics committee of our institution and selected 30 patients with prostate cancer who underwent moderate‐hypofractionated radiotherapy (before and after the introduction of Lilium). Table 1 lists patient characteristics. The patients were advised to collect urine approximately 1–2 h prior to the CT simulation and to drink a cup of water (approximately 200 ml), and they were immobilized with an evacuate cushion in a supine position. A dual‐energy CT scanner (Revolution HD; GE Medical Systems, Milwaukee, WI, USA) was used for image acquisition using the following scanning parameters: tube voltage, 140/80 kVp; tube current, 375 mA; helical pitch, 0.984:1.

**TABLE 1:** Patient characteristics

	Without Lilium	With Lilium
Number of patients, (*n*)	15	15
Age (years), median (range)	69 (50–79)	69 (47–81)
Treatment plan		
Prescribed dose (45/54/55/60 Gy), (*n*)	0/0/1/14	1/2/1/11
Number of fractions (15/18/20 fractions), (*n*)	0/0/15	1/2/12
Clinical target volume (prostate/prostate + half seminal vesicle/prostate + seminal vesicle), (*n*)	4/11/0	2/11/2
Planning target volume (ml), median (range)	62 (42–90)	68 (44–163)
Planning target volume overlapped with bladder (ml), median (range)	4 (2–6)	5 (2–12)
Bladder volume (ml), median (range)	137 (48–506)	151 (95–315)
Percent volume of bladder receiving 40 Gy (%), median (range)	11 (3–22)	10 (4–20)
Percent volume of bladder receiving 35 Gy (%), median (range)	14 (4–30)	12 (5–24)
Percent volume of bladder receiving 30 Gy (%), median (range)	18 (4–43)	17 (7–30)

The CT images were reconstructed with a slice thickness of 2 mm and transferred to a treatment planning system (Eclipse; Varian Medical Systems, Palo Alto, CA). A clinical target volume (CTV) was defined as the prostate gland ± seminal vesicles, and an isotropic margin of 4 mm was added to the CTV to generate the planning target volume (PTV). A prescription dose of 45–60 Gy was delivered using VMAT technique to the PTV in 15–20 fractions (2.75‐3 Gy/fraction). Varian C‐arm treatment units with six degrees of freedom couches were used for dose delivery (Varian Medical Systems, Palo Alto, CA, USA). For treatment, 15 patients (without Lilium group) collected urine before approximately 1–2 h prior to treatment and drank a cup of water as in the simulation. Subsequently, they were immobilized just as in the simulation, and daily CBCT images were acquired before dose delivery for the target localization. The spatial integrity of the images acquired by the dual‐energy CT scanner and CBCT was tested periodically using CATPHAN 600 phantom (Phantom Laboratory, Salem, NY), in which the air and Teflon rods are spaced in a given distance (50 mm). The phantom was chosen because it equips various modules that can test CT number uniformity, spatial resolution, and so on.^13^

### 2.2 BV measurements using Lilium ultrasound scanner

**FIGURE 1:**
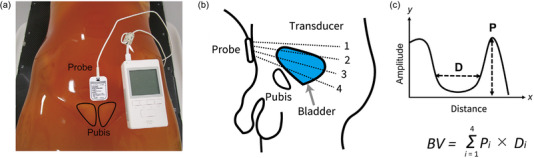
(a) Photograph of Lilium® α‐200 portable ultrasound bladder scanner (Lilium Otsuka, Kanagawa, Japan). (b) Bladder volume measuring approach. (c) Calculation formula for bladder measurement

For 15 patients, Lilium was used to quantitatively measure the BV in the CT simulation and treatment (with the Lilium group). A small ultrasound probe attached to the Lilium was placed on the suprapubic region of the patient in a supine position (Figure 1a). The probe was equipped with four ultrasonic transducers (Figure 1b), and the distance and amplitude of the ultrasound wave were represented on the x‐ and y‐axis, respectively, using the A‐mode technique (Figure 1c). BV was estimated based on the ultrasound waveform obtained from the four transducers as follows:

$$\mathrm{BV}=\sum_{i=1}^{4}P_{i}\times D_{i},$$

where *P_i_* and *D_i_* indicate amplitude and distance between the two graphical spikes in the amplitude of the ultrasound wave (anterior and posterior bladder wall). For treatment, BV was measured using the Lilium before CBCT acquisitions. In case the BV was less than 80% of that in the CT simulation, the patient was advised to drink more water and wait, and the measurement of BV using Lilium was repeated before treatment until the desired BV was achieved. However, in cases where the patients expressed intolerability of uresiesthesia, CBCT was performed, and doses were delivered accordingly. All BV measurements were performed by well‐trained radiation therapists.

### 2.3 Data analysis

The daily BVs were measured using CBCT images and Lilium. Pearson's correlation coefficient was used to measure the strength of the linear relationship between the BVs measured by CBCT and Lilium. The absolute value of Pearson's *r* was defined as very weak (0–0.19), weak (0.2–0.39), moderate (0.40–0.59), strong (0.6–0.79), and very strong (0.8–1). The linear regression analysis was also performed to obtain a regression equation. Subsequently, the relative daily BV ratio for each patient was defined as the ratio of BV in the treatment and simulation. The daily variation of dosimetric parameters for the bladder was assessed based on the dose distribution in the treatment plan, and the BVs contoured using CBCT. The daily volumes of bladder receiving a specific dose in the range of 30–40 Gy (V_30Gy_‐V_40Gy_), which were predictive indicators of genitourinary toxicity,^14^, ^15^ were evaluated and compared with those in the treatment plan. The absolute difference in the dosimetric parameters between the groups with and without Lilium was compared using the Mann–Whitney *U* test. Statistical significance was set at *p* < 0.05. All statistical analyses were performed using the SPSS software (version 27; IBM, Armonk, NY).

## 3 RESULTS

**FIGURE 2:**
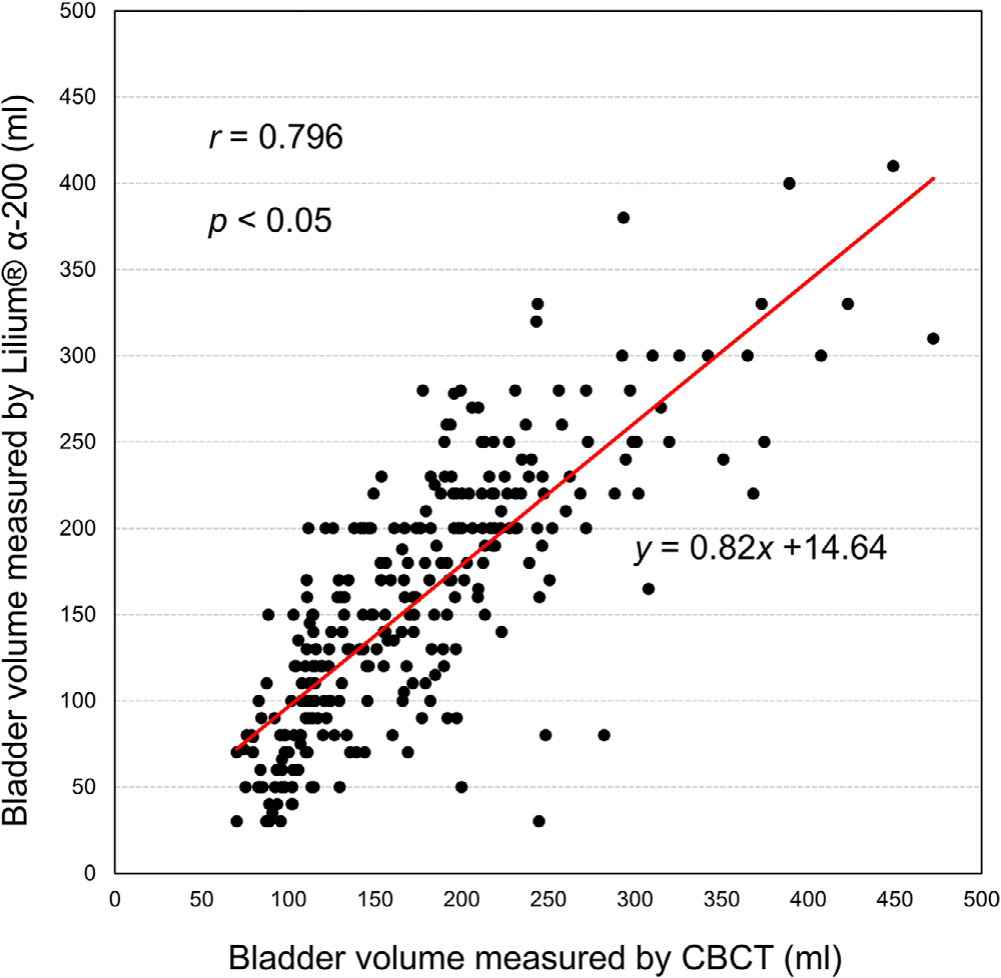
Relationship of bladder volume between that measured by cone‐beam computed tomography (CBCT) and Lilium® α‐200

A patient discontinued treatment leaving one treatment session, and a BV could not be measured using Lilium due to malfunction. Consequently, 289 pairs of BVs measured using CBCT and Lilium were analyzed for the Lilium group. Figure 2 shows the relationship between the BVs measured using CBCT and Lilium. The BVs measured using Lilium increased as measured using CBCT also increased. There was a strong positive relationship (*r* = 0.796, *p* < 0.05) between the BVs using the two devices.

**FIGURE 3:**
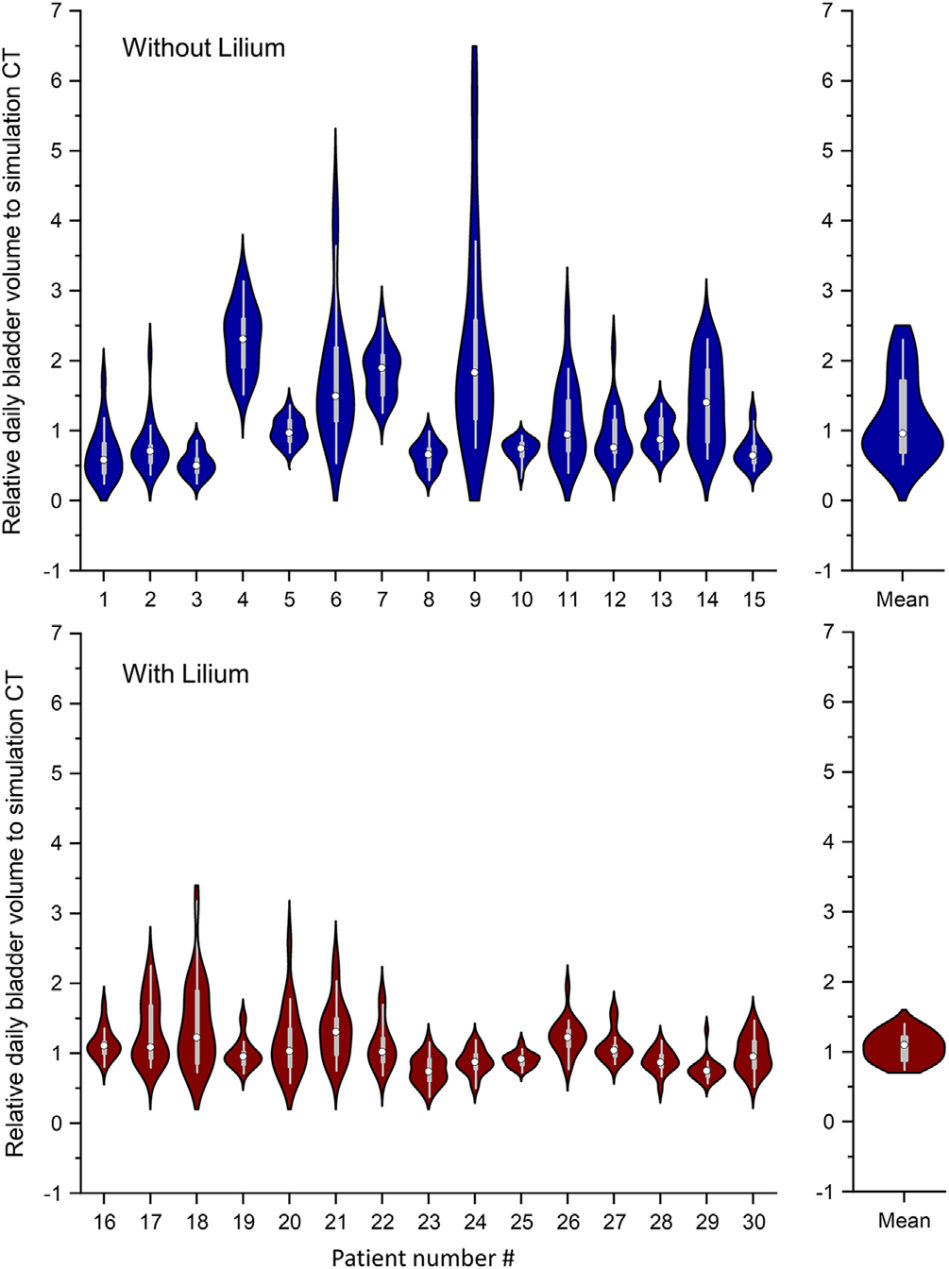
Daily variation of bladder volumes (BVs) measured using cone‐beam computed tomography (CBCT) without and with Lilium® α‐200 groups for each patient. Boxes denote median value and upper and lower quartiles; whiskers denote maximum and minimum values within 1.5 × inter‐quartile range

The daily variation of BVs with and without Lilium groups is shown in Figure 3. A large variation in BV was observed when Lilium was not used, and the relative daily BV ratio to simulation CT ranged from 0.26 to 6.22. Without the Lilium group, relative BV ratios of < 0.5 and > 2 were observed in 10.3% and 12.7% of treatment sessions, respectively, and only 22.7% of treatment sessions were within 0.8–1.2. By contrast, the daily variation in BV was reduced with the Lilium group, and the minimum and maximum relative BV ratio was 0.39 and 3.19, respectively. The relative BV ratios of < 0.5 and > 2 were observed at 1% and 2.8% of treatment sessions, respectively. Moreover, 56.4% of treatment sessions were within 0.8–1.2.

Table 2 summarizes the absolute difference in dosimetric parameters between the bladder in the treatment plan and daily CBCT. The mean absolute difference in dosimetric parameters in all evaluated dose regions was large when Lilium was not used. The respective mean ± standard deviation (SD) of absolute difference in V_30Gy_ (6.7% ± 6.3%), V_35Gy_ (5.1% ± 4.7%), and V_40Gy_ (4.0% ± 3.6%) without Lilium group were significantly larger (*p* < 0.05) than those with Lilium (3.7% ± 2.8%, 2.9% ± 2.2% and 2.4% ± 1.8% for V_30Gy_, V_35Gy_, and V_40Gy_, respectively).

**TABLE 2:** Absolute difference in dosimetric parameters between bladder in the treatment plan and daily CBCT

Dosimetric parameter	Without Lilium		With Lilium		
(%)	Mean	SD	Mean	SD	*p*‐value
V_40Gy_	4.0	3.6	2.4	1.8	<0.05
V_35Gy_	5.1	4.7	2.9	2.2	<0.05
V_30Gy_	6.7	6.3	3.7	2.8	<0.05

## 4 DISCUSSION

In this study, we report the advantage of using a newly developed A‐mode portable ultrasound bladder scanner to reduce daily variation in BV during a moderate‐hypofractionated VMAT treatment course for patients with prostate cancer. Yee et al. analyzed BV using the daily CBCT images for patients with bladder cancer and found significant variations in bladder and PTV volume and position occurred.^16^ Chen et al.^8^ reported the dosimetric impact of different bladder filling during prostate cancer radiotherapy and found that a 10% increase in BV causes a 5.6% decrease in the mean dose. Moreover, Mak et al. reported considerable interfractional displacement of seminal vesicles in prostate radiotherapy, and the displacement was correlated with bladder filling.^17^ Therefore, maintaining BV consistency is imperative for reducing doses to the bladder and ensuring accurate dose delivery.

The ultrasound scanner has been utilized as a simple and noninvasive BV measurement, and measurement in a supine position is considered as a gold standard.^18^ Currently, several portable ultrasound bladder scanners that can measure BV with adequate accuracy are clinically available. Yoon et al. used a portable three‐dimensional ultrasound scanner (Biocon‐700; Mcube Technology, Korea), in which the ultrasound transducer was rotated 120° to obtain 12 images of B‐mode. A strong correlation existed between the BV measured using the bladder scanner and planning CT.^12^ Another report by Luo et al. also demonstrated the adequate accuracy of the BV measurements using a portable bladder scanner (Bladder‐Scan BVI9400; Verathon Medical, The Netherlands) in which the internal transducer moved 360° to scan 12 planes to produce a three‐dimensional B‐mode image.^11^The use of biofeedback protocol with the B‐mode portable ultrasound scanner is an easy to use and is an effective approach to reduce daily BV variations.^19^ In our study, a more simplified portable bladder scanner utilizing the A‐mode technique was used to measure the BV, and a significantly strong correlation was observed between the BV measured by the Lilium ultrasound scanner and CBCT images (Figure 2). BV measured using CBCT was determined as the external line of the bladder wall to evaluate dosimetric parameters of bladder in the treatment plan, while the BV measured using Lilium was calculated based on the inside line of the anterior bladder wall and midline of the posterior bladder wall (Figure 1). Thus, in this study, BV was underestimated in the A‐mode device compared to CBCT. Compared with B‐mode ultrasound devices, which map soft tissues in a two‐dimensional image, A‐mode devices are generally more cost‐effective and have less reliance on a technician's skill, training, and time.^20^ However, we should be aware that the accuracy of the BV measurement using the Lilium was more affected by the patient's characteristics than that using the B‐mode device. Yamaguchi et al. reported that flattened bladder shape and large prostates could be factors in measurement error in the BV measurement using the Lilium.^21^ In fact, Luo et al. demonstrated the stronger linear relationships between the BV measured using B‐mode device and CBCT (*r* = 0.91) compared with that obtained in our study (*r* = 0.796).^11^ Because scanner malfunction can be a source of measurement error, a quality assurance (QA) program is essential for proper operation of the ultrasound scanner. Hykes et al. described the QA program for A‐mode real‐time ultrasound scanner to maintain long‐term performance, and the transducers were recommended to be tested at least monthly using a phantom with various patterns of rods (AIUM 100 mm Standard Test Object).^22^ Ideally, the QA program needs to be performed using a tissue‐equivalent phantom, which exhibits the same behavior (velocity, reflection scattering, absorption, and attenuation) with human anatomy. However, the Lilium ultrasound scanner cannot utilize the commercially available phantom, and the QA depends on maintenance by a service from the manufacturer. A phantom to test the spatial integrity of the Lilium ultrasound scanner should be developed, and the QA program needs to be performed periodically by users.

The bladder is a highly distensible organ, and its position can move due to respiratory motion and bowel filling. Thus, the dosimetric parameter for the bladder in treatment planning is not accurate, and it is difficult to understand the actual delivered doses.^23^ In fact, few studies have reported true three‐dimensional bladder dosimetry in relation to toxicities in prostate radiotherapy. In our study, the absolute differences in dosimetric parameters between the BVs in simulation CT and daily CBCT were significantly reduced (Table 2), suggesting that the A‐mode portable ultrasound scanner can be an effective tool for improving the reliability of dosimetric parameters in the treatment plan. A single institutional DELINEATE trial showed that cumulative late radiation therapy oncology group grade >2 genitourinary toxicity at 1 year was observed in 0% and 10% of the standard (74 Gy in 37 fractions) and moderate‐hypofractionated (60 Gy in 20 fractions) prostate radiotherapy, respectively.^24^ Thus, without rigorous management of BVs during hypofractionated prostate radiotherapy, unexpected radiation side effects may occur.^7^

This study has some limitations as well. First, the number of patients was limited, and depth analysis could not be performed, such as the relationship between BV reproducibility and age. Second, BV was measured using Lilium only before treatment in this study; however, it was continuously changing. Nathoo et al. measured the BV using a portable diagnostic ultrasound scanner at 15‐min intervals over 60 min and generated the individual bladder kinetic model for ensuring the reproducible BV. They concluded that the optimal urine correction is 60 min after voiding and drinking 500 ml of water.^25^ Lotz et al. found a large variation in the urine inflow rate between individuals, ranging from 2.1 to 15 ml/min, and it was linearly correlated with age (negative slope).^26^ Real‐time monitoring of BV has the potential to improve the reproducibility of BV during treatment. The A‐mode portable bladder scanner used in this study can measure BV continuously, while the B‐mode devices require medical staff to provide BV measurements at regular intervals. The features of the A‐mode device may improve the reproducibility of BV with the minimal workload in clinical radiotherapy. Third, the daily dosimetric parameter of the bladder was estimated based on the dose distribution on the planning CT image, while the patient's position and anatomical changes of OARs were not accounted for. Finally, Lilium was introduced only for patients with prostate cancer, although the other treatment sites in the pelvic region (such as cervical cancer and rectal cancer) also require a full bladder.^27^, ^28^ Despite these limitations, our study demonstrates the potential of the A‐mode portable bladder scanner for reducing the variation in BV during a treatment course and provides useful information for maintaining a full bladder.

## 5 CONCLUSION

The BV measured by Lilium was significantly correlated with that measured by CBCT. The use of this portable ultrasound bladder scanner could improve the reproducibility of daily BV and reduce the dosimetric deviation between the simulation and treatment. The A‐mode portable ultrasound scanner (Lilium) has potential to be a screening tool for the daily BV measurement prior to performing CBCT with feasible accuracy to maintain the BV during treatment course for prostate cancer patient.

## CONFLICT OF INTEREST

The authors have no conflict of interest to declare in relation to this study.

## AUTHOR CONTRIBUTIONS

*Concept and design*: Shingo Ohira and Riho Komiyama, Masashi Nakayama, Kayo Sakai, and Takero Hirata. *Bladder volume measurement*: Shingo Ohira, Riho Komiyama, Kento Yoshikata, and Yoshihiro Ueda. *Data analysis*: Shingo Ohira and Riho Komiyama. *Manuscript preparation*: Shingo Ohira, Riho Komiyama, Naoyuki Kanayama, Kayo Sakai, Takero Hirata, Kento Yoshikata, Yoshihiro Ueda, Masayoshi Miyazaki, Masashi Nakayama, Masahiko Koizumi, and Koji Konishi. All authors read and approved the final manuscript.
